# Validation of a Novel Diagnostic Approach Combining the VersaTREK™ System for Recovery and Real-Time PCR for the Identification of *Mycobacterium chimaera* in Water Samples

**DOI:** 10.3390/microorganisms9051031

**Published:** 2021-05-11

**Authors:** Roberto Zoccola, Alessia Di Blasio, Tiziana Bossotto, Angela Pontei, Maria Angelillo, Alessandro Dondo, Maria Goria, Simona Zoppi

**Affiliations:** 1 Istituto Zooprofilattico Sperimentale del Piemonte, Liguria e Valle d’Aosta, 10154 Torino, Italy; roberto.zoccola@izsto.it (R.Z.); tiziana.bossotto@izsto.it (T.B.); angela.pontei@izsto.it (A.P.); maria.angelillo@izsto.it (M.A.); alessandro.dondo@izsto.it (A.D.); maria.goria@izsto.it (M.G.); simona.zoppi@izsto.it (S.Z.); 2 Azienda Sanitaria Locale TO3 S.C. Sanità Animale, Pinerolo, 10064 Torino, Italy

**Keywords:** non-tuberculous mycobacteria, culture media, limit of detection, real-time polymerase chain reaction

## Abstract

*Mycobacterium chimaera* is an emerging pathogen associated with endocarditis and vasculitis following cardiac surgery. Although it can take up to 6–8 weeks to culture on selective solid media, culture-based detection remains the gold standard for diagnosis, so more rapid methods are urgently needed. For the present study, we processed environmental *M. chimaera* infected simulates at volumes defined in international guidelines. Each preparation underwent real-time PCR; inoculates were placed in a VersaTREK™ automated microbial detection system and onto selective Middlebrook 7H11 agar plates. The validation tests showed that real-time PCR detected DNA up to a concentration of 10 ng/µL. A comparison of the isolation tests showed that the PCR method detected DNA in a dilution of ×10^2^ CFU/mL in the bacterial suspensions, whereas the limit of detection in the VersaTREK™ was <10 CFU/mL. Within less than 3 days, the VersaTREK™ detected an initial bacterial load of 100 CFU. The detection limit did not seem to be influenced by NaOH decontamination or the initial water sample volume; analytical sensitivity was 1.5 × 10^2^ CFU/mL; positivity was determined in under 15 days. VersaTREK™ can expedite mycobacterial growth in a culture. When combined with PCR, it can increase the overall recovery of mycobacteria in environmental samples, making it potentially applicable for microbial control in the hospital setting and also in environments with low levels of contamination by viable mycobacteria.

## 1. Introduction

*Mycobacterium chimaera*, a slow-growing mycobacterium of the *Mycobacterium avium-intracellulare* complex (MAC) was first identified in 2004 [1]. It was often misidentified as *Mycobacterium intracellulare* until the 16–23S internal transcribed spacer (ITS) region was sequenced [2]. Like Mycobacterium avium complex members, *M. chimaera* is commonly isolated from water, where it can produce biofilms that are difficult to remove from surfaces and water pipes [3]. Its prevalence in the environment is largely unknown in Europe [4,5], yet it is a well-known opportunistic bacterium responsible for severe lung infection, especially in the immunocompromised or the elderly with chronic respiratory diseases [6].

*M. chimaera* has attracted growing interest since 2011 with the increased incidence of endocarditis and vasculitis after cardiac surgery [7,8]. Surveys following the first outbreaks in Switzerland and the Netherlands identified contaminated heater–cooler units (HCUs) as the source of the airborne transmission of *M. chimaera* through the aerosolization of water in surgical suites during open chest surgery [2,7]. Extensive molecular investigations during the outbreaks pinpointed a single common source of *M. chimaera*, leading to the hypothesis that contamination occurred in the LivaNova HCUs during manufacture [9] in European countries, including Italy [2]. Since then, other *M. chimaera* strains have been isolated from other HCU devices [2], although these are genetically different from those involved in the first outbreaks and not associated with human clinical cases [10].

Clinically, human infection by *M. chimaera* is characterized by a long latency period (up to 6 years), disseminated forms, and a high mortality rate [2]. Intrinsic resistance against disinfectants and antibiotics makes recovery unlikely [11]. Since 2013, more than 100 cases of HCU device-related *M. chimaera* infection have been reported [2,10]. Given the health implications of this nosocomial problem, the scientific community of the European Centre for Diseases and Control and Public Health England have established diagnostic protocols to detect *M. chimaera* in water and air samples from HCUs [7,12,13].

Since it is a slow-growing mycobacterial species, *M. chimaera* can take up to 6–8 weeks to culture on selective solid media and liquid culture could guarantee better performance and reduce the time for recovery [14]. Culture-based methods of detection remain the gold standard for diagnosis, however, so there is an urgent need for methods that are rapid and reliable [2].

Several recent methods utilizing liquid media for recovering mycobacteria have demonstrated a reduction in the time to detection (TTD) of targeted bacteria [15].

Veterinary laboratories are well acquainted with the diagnostic aspects of mycobacteriosis, its role in animal diseases and zoonoses [16,17], and the use of the VersaTREK™ automated microbial detection system (Thermo Fisher Scientific, Waltham, MA, USA) in MAC infection detection [18]. The VersaTREK™ system is a fully automated, continuous system for monitoring the growth and detection of several types of bacteria, including mycobacteria, depending on the cultural media used. The technology is based on the detection of pressure changes due to either the production or the consumption of gas inside the headspace of the medium bottles. Microbiological studies in human medicine have reported a considerably shorter TTD with liquid media compared to solid media [19,20]. Chien et al. [21] reported that the TTD for mycobacteria recovery was 10.7 days with a BACTEC™ MGIT™ 960 instrument (Beckton-Dickinson, Franklin Lakes, NJ, USA) compared to 30.6 days with a Löwenstein–Jensen (LJ) medium.

Since the year 2000 [22,23,24,25,26,27], the diagnostic laboratories of the Istituto Zooprofilattico Sperimentale del Piemonte, Liguria e Valle d’Aosta (IZSPLV) have been involved in the bovine tuberculosis eradication program and in reducing the prevalence of paratuberculosis. These efforts have led to a standardized protocol based on the isolation and molecular characterization of bacterial strains of veterinary interest in the genus *Mycobacterium*. Given the genetic affinity between *M. chimaera* and other MAC strains routinely isolated, a diagnostic flowchart has been optimized for the rapid detection of environmental contamination by *M. chimaera* by means of the VersaTREK™ automated system based on mycobacteria specific liquid media.

In the present study, we describe a novel diagnostic approach that can reduce the time to recover and identify *M. chimaera*. We also set out its limits of detection (LOD) using the VersaTREK™ system and a real-time PCR which is currently in use at the IZSPLV laboratories.

## 2. Materials and Methods

The *M. chimaera* NCTC 13781 (DSM 44623) strain, purchased from the collection of the Public Health England (Porton Down, Salisbury, UK), was used for all tests.

### 2.1. Part One: Fine-Tuning of Bacteriological and Molecular Methods for Detection of M. chimaera

A suspension (McFarland standard 3) of approximately 9*× 10^8^CFU/mL, was prepared [28] and a logarithmic dilution series was created by adding 1 mL of the undiluted suspension to 9 mL of VersaTREK™ Myco Media (modified Middlebrook 7H9 broth, Thermo Fisher Scientific; mMVT) and phosphate buffered saline (PBS) added with 0.5% Tween 20 (PBS-T, Merck Life Science srl, Milan, Italy). Two sets of eight serial dilutions to 9 CFU/mL were prepared, lettered from “A” to “I”, and then each assessed in a culture and molecular assay.

#### 2.1.1. Culture Methods of *M. chimaera* Isolation

Ten microliters of each suspension were seeded on 7H11 selective solid media (Liofilchem srl, Roseto degli Abruzzi, Italy; s7H11) supplemented with 10% oleic albumin dextrose catalase (OADC, Liofilchem), polymyxin B (200,000 IU/L final concentration, *Bacillus cereus* supplement, Liofilchem) and a ready-to-use supplement (*Campylobacter* cefoperazone, trimethoprim, vancomycin, amphotericin B (CTVA), Liofilchem) composed of amphotericin B (10 mg/L final concentration), trimethoprim (20 mg/L final concentration), cefoperazone (20 mg/L final concentration), vancomycin (20 mg/L final concentration) in triplicate and incubated at 37 °C ± 1.5 for 8 weeks or until positive. Cultures were assessed weekly; the growth of suspected scotochromogen and rough colonies were identified by PCR.

Suspensions were tested in triplicate with the VersaTREK™ system according to the manufacturer’s instructions. First, 1 mL was inoculated into flacons containing mMVT and sponges that provide support for growth and increase the surface area was exposed to headspace oxygen. We then added 1 mL of VersaTREK™ Myco growth supplement (Myco GS). After rapid shaking, the spiked cultures were placed into the VersaTREK™ instrument and incubated at 37 °C ± 1.5. During incubation, the system continuously monitors (84 days) changes in either gas production or gas consumption in the bottles. A “knee shaped” mycobacterial growth curve is generated by a specific algorithm. The instrument gives a positive signal at approximately ×10^6^ CFU/ mL, at which point the bottle is removed from the instrument, vigorously shaken to dislodge microorganisms from the sponge, and the culture media is aspirated with a syringe. After centrifugation at 3000 g for 15 min, the supernatant is removed, and the pellet re-suspended in PBS and streaked on s7H11 agar plates; growth is assessed weekly and suspected colonies are identified by PCR.

#### 2.1.2. Setting and Validation of Molecular Methods for *M. chimaera* Detection

Aliquots (0.5 mL) of the suspensions were tested by biomolecular assay. DNA extraction was performed by heat treatment. The real-time PCR protocol was adapted from Zozaya-Valdés et al. [29] to detect a 79 bp fragment of the SR1 region, identified as highly specific for *M. chimaera*. The SR1 region target for the TaqMan assay was selected by aligning 96 mycobacterial genome sequences, including 63 *M. chimaera* sequences from different countries, and it was 100% conserved among all *M. chimaera* templates. The protocol was optimized in 10 µL using iTaq Universal Probes Supermix (Bio-Rad Laboratories srl, Segrate, Italy) with a CFX384 Touch real-time PCR detection system (Bio-Rad Laboratories) conducting 40 cycles at 95 °C for 10 s and at 60 °C for 20 s.

Analytical sensitivity of the real-time PCR was measured, starting from extracted DNA brought to a concentration of 100 ng/µL and then diluted in base 10 to a concentration of 1 pg/µL. Assay specificity was tested using DNA extracted from 22 different bacterial species (11 of the genus *Mycobacteria* were derived from international collections -ATCC). In particular, *Mycobacterium intracellulare* corresponded to ATCC 35847. Other bacterial strains were characterized by Sanger sequencing of the 16s rRNA region.

#### 2.1.3. Statistical Analysis

Normality of data distribution was tested with the Shapiro–Wilk test. A Two-Way ANOVA was applied to assess the effects of suspension concentrations (A–I) and liquid media (mMVT vs PBS-T) on positivity time of detection followed by Bonferroni’s post-hoc test. Suspension concentrations and liquid media, and suspension concentration × liquid media interaction were independent variables. Data are reported as mean ± standard deviation (SD). The criterion for significance was set at *p* < 0.05. Statistical analysis was performed using GraphPad Prism (GraphPad Software, San Diego, CA, USA).

### 2.2. Part Two: Fine-Tuning of Detection of M. chimaera from Decontaminated Water Samples

A stock solution of 0.5 McFarland using *M. chimaera* NCTC 13781 (DSM 44623) strain was prepared to a final concentration of 1.5 × 10^8^CFU/mL.

#### 2.2.1. Inoculation and Decontamination of 1000 mL Water Sample

A logarithmic dilution series was created by adding 10 mL of the undiluted suspension to 900 mL of deionized sterile water until 1.5 × 10^2^ CFU/ mL for a total of five samples, lettered from “a” to “e”.

A specific protocol was applied based on national (AMCLI-GLAMIC) and international (ECDC) guidelines [7,30]. Briefly, water was filtered using 0.45 um pore filters in cellulose nitrate, which were then fragmented and placed in 2 mL of mMVT. Decontamination was performed with natrium hydroxide (NaOH) 2% at a ratio of 1:1, vortexed for 20–30 s, left for 15–20 min at room temperature and vortexed every 5 min.

#### 2.2.2. Inoculation and Decontamination of 100 mL Water Sample

A logarithmic dilution series was created by adding 1 mL of the undiluted suspension to 90 mL of deionized sterile water to 1.5 × 10^2^ CFU/ mL for a total of five samples, lettered from “a” to “e”.

A specific protocol was applied following Public Health England (PHE) guidelines [13]. Briefly, samples were split into two aliquots of 50 mL each and centrifuged at 3000× *g* for 15 min. The supernatant was removed, leaving approximately 1 mL and the two aliquots were pooled. Decontamination was performed by adding an equal volume of 4% NaOH, mixing by vortex for 20–30 s, then left for 15–20 min at room temperature and vortexed every 5 min.

## 3. Results

### 3.1. Part One: Bacteriological Detection of M. chimaera

Using the s7H11 solid media, *M. chimaera* was detected only in suspensions A and B prepared with PBS-T (Table 1). In contrast, the VersaTREK™ system returned a positive signal for all tested suspensions; the result was confirmed due to the growth in *M. chimaera* by culture and real-time PCR. Based on these data, <10 CFU/ mL may be assumed as the LOD for the VersaTREK™ system (Table 1).

**Figure 1:**
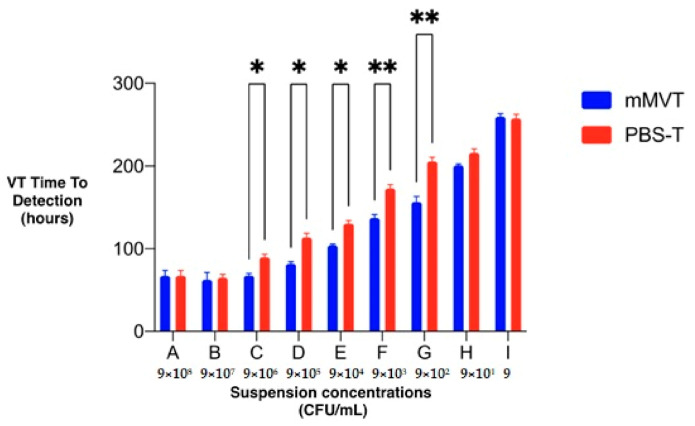
Bonferroni’s post-hoc test revealed a significant difference in positivity time of detection between suspension concentrations (A–I) and liquid media (mMVT vs PBS-T). Bars (mean ±standard deviation) indicate the positivity time for detection of *M. chimaera* by VT and asterisks indicate significant differences (* *p* < 0.05; ** *p* < 0.01). mMVT denotes VT Myco Media (modified Middlebrook 7H9 Broth, Trek Diagnostic System, Thermo Fisher Scientific); PBS-T phosphate-buffered saline added with 0.5% Tween 20.

Threshold cycles obtained with real-time PCR showed that the results of each serial dilution detached from the previous one after about 3.3 cycles. This evidence, related to the PCR mechanism, doubled the target at each cycle (and decoupled in 3.3 cycles), confirming the correctness of the starting suspensions. Our data show that the time for a positive signal from the VersaTREK™ system ranged between 2.3 and 10.9 days (or 55.2 h and 262 h, respectively). The mean positivity time of detection was significantly affected by the liquid media (F 372.43, DFn 1, DFd 4, *p* < 0.0001) and the suspension concentration (F 1184.93, DFn 8, DFd 32, *p* < 0.0001). The suspension concentration had the same effect at all values of liquid media (F 19.52, DFn 8, DFd 32, *p* < 0.0001). Bonferroni’s post-hoc test revealed a significant difference in the positivity time of detection between the two liquid media for suspension concentrations C–G (Figure 1).

**Figure 2:**
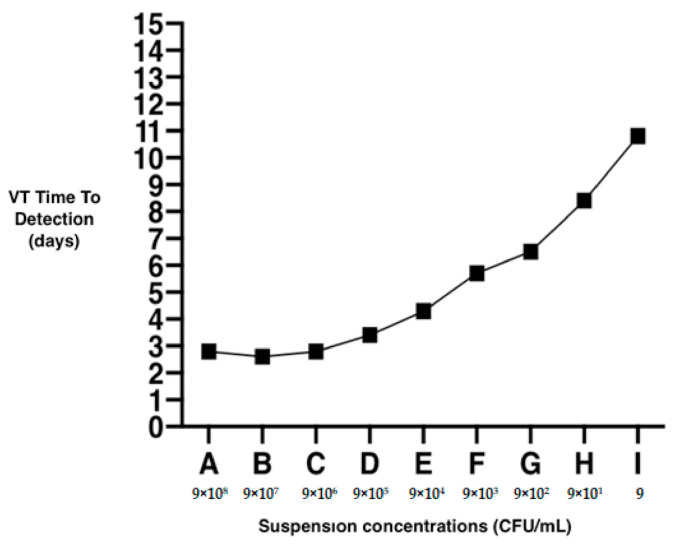
Time rate of exponential growth (one log) for concentrations (A–I) after incubation in the VersaTREK™ system and using mMVT broth.

As indicated by the manufacturer, the VersaTREK™ system can detect mycobacterial growth when the bacterial load in the bottle reaches ×10^6^ CFU/mL, taking at least 3 days to generate a first positive signal. Accordingly, for samples A, B, and C, where the initial concentration was ≥×10^6^ CFU/mL, the TTD was around 3 days. A time rate of exponential growth, corresponding to 1 log, was about every 2 days (±SD 2.5 days) for the other suspensions (from D to I) as shown in Figure 2.

### 3.2. Part One: Validation of Molecular Methods for M. chimaera Detection

Validation tests of the real-time PCR assay showed that it detected *M. chimaera* DNA up to a concentration of 10 pg/µL. Comparison with the isolation tests showed that the biomolecular method detected the DNA of the bacterial suspensions down to a dilution of 9 × 10^2^ CFU/mL (Table 1).

Analytical specificity tests showed that real-time PCR did not detect positivity in 21 of the 22 (95.45%) bacterial species tested (Table 2).

Weak positivity was found only for *Mycobacterium intracellulare* with very high threshold cycles (Ct > 38).

### 3.3. Part Two: Detection of M. chimaera from Decontaminated Water Samples

When we tested the different volumes of inoculums, we recovered *M. chimaera* from all suspensions prepared in 1000 mL. Positivity was detected in all suspensions prepared in 100 mL with liquid media and only in the first four suspensions (A-B-C-D) with the solid media (Table 3).

### 3.4. Molecular Methods for the Detection of M. chimaera from Decontaminated Water Samples

Molecular analyses performed on the inoculum material detected *M. chimaera* DNA in all suspensions prepared in 1000 mL with solid and liquid media, while positivity was detected only in the first three suspensions (A-B-C) prepared from 100 mL (Table 3).

## 4. Discussion

The setting of bacteriological methods for the detection of *M. chimaera* was conducted using solid (s7H11) and liquid (mMVT and PBS-T for VersaTREK™) media. Our observation of the higher performance for the liquid media is shared by previous studies [14,15,18]. As also observed in Table 1, while the s7H11 medium can efficiently recover *M. chimaera*, as reported elsewhere [18], it tends to form clumps, leading to an uneven bacterial concentration in the samples. The use of PBS-T as a liquid suspension seems to be useful only at high concentrations (Table 1) in comparison to mMVT, even if not enough data are available to prove it. In addition, the dilution effect needs to be taken into consideration because, compared to 1 mL inoculum in a VersaTREK™ bottle, the routine use of 10 μL of suspension for culturing on solid media can negatively affect *M. chimaera* growth and detection. Contrastingly, in the environmental samples where the entire volume was centrifuged (100 mL) or filtrated (1000 mL) all viable bacteria were recovered, thus enhancing the performance of the s7H11 medium (Table 3) while overcoming the problem of clumping and the dilution effect from bacterial concentration.

A similar observation could explain the failure of the real-time PCR to detect DNA at higher dilutions (Table 1 and Table 3): clump formation and dilution (aliquot of 0.5 mL) may not ensure the detection of *M. chimaera* DNA at such concentrations. Conversely, the VersaTREK™ system appears to be more sensitive in detecting viable *M. chimaera* in samples with a very low concentration (Table 1 and Table 3). Analytical sensitivity is higher in a liquid cultural system than in a solid one [15,18,19,20,21,23,31,32,33,34,35,36,37], which is why the use of an appropriate liquid medium is crucial. Our data show that the use of mMVT as a liquid base for suspensions seems to guarantee a better performance of the VersaTREK™ in terms of positivity time. Since the mMVT is a component of the VersaTREK™ kit, an active role could be hypothesized for growth in *M. chimaera* in the VersaTREK™ system.

Real-time PCR detected *M. chimaera* DNA up to a concentration of 10 pg/μL; this result is comparable with the sensitivity (two genomic copies) reported by Zozaya-Valdés et al. [29]. A DNA search using PCR will detect dead as well as live bacteria, however. PCR can be a useful tool for the fast identification with a *M. chimaera*-specific PCR assay and while it provides a rapid yes/no result, it is indissoluble with cultural methods for assessing biological risk.

Molecularly weak positive results obtained with *M. intracellulare* during specificity tests can be explained by the close phylogenetic proximity between the two bacterial species [2]. As suggested by the European Center for Disease Prevention and Control, *M. chimaera* identification should be performed by sequencing at least two conserved fragments (16S-23S rRNA ITS, 16S rRNA, rpoB, hsp65) to increase specificity. They also suggest that the test could be further developed by inserting more DNA targets. This was not done in the present preliminary test; further studies are needed to improve specificity.

The VersaTREK™ system, by virtue of its fully automated continuous monitoring, can recognize mycobacteria growth and return a positive signal when the bacterial load in the bottle reaches 10^6^ CFU/mL. Based on the positivity time recorded in our tests, the samples spiked one log approximately every 2 days of incubation. The increase in cell numbers over a given time interval can be considered proportional to the number of cells at the start of the incubation time. Our data may be useful for defining an initial load of environmental samples proportional to the time required for positivity.

Several authors reported the efficient use of the VersaTREK™ system in promptly recovering mycobacteria [15,18], including strains belonging to MAC [18]. To the best of our knowledge, the use of the VersaTREK™ as a tool to detect *M. chimaera* in water samples has been described [36] but not investigated in detail. In our study, we can confirm the good performance in recovering mycobacteria and suggest that the detection limit of the VersaTREK™ system did not seem to be influenced by the initial volume of the water samples. A comparison between the VersaTREK™ and other automated liquid media for mycobacterial culture and a comparison of the two water sample volumes (100 mL and 1000 mL) showed no differences in the detection limit of *M. chimaera*, in contrast with findings reported by Schreiber et al. [37]. The sensitivity of the VersaTREK™ instrument for detecting *M. chimaera* in NaOH decontaminated filtered 1000 mL water samples and centrifuged 100 mL water samples overlapped by about 150 CFU or less. The use of 2% NaOH as a decontamination agent, as used for *M. bovis* recovery from animal tissues [23] and as reported in other previous studies on the Mycobacterium avium complex [31,33,34], did not minimize recovery of the target bacteria, but was the best solution for the VersaTREK™ system (unpublished data).

Moreover, cetylpyridinium chloride (CPC) is considered the most effective agent for the successful recovery of mycobacteria, including nontuberculous mycobacteria in water samples [35]. It is not reliable with the VersaTREK™ system, however, because it does not allow the automatic system detection to function properly probably due to its surfactant property (unpublished data). Before using CPC, decontamination in half brain heart infusion broth should be performed, as reported for the *Mycobacterium avium subsp. paratuberculosis* procedure in a VersaTREK™ system [18], followed by centrifugation prior to inoculation. This may cause a potential loss of viable bacteria and is an unnecessarily time-consuming procedure that can easily and efficiently be replaced by NaOH 2%.

## 5. Conclusions

In conclusion, our data show that the integrated diagnostic protocol provides a rapid response, with sufficient analytical sensitivity and specificity, making it potentially applicable for controlling the effectiveness of environmental and instrumental sanitization in hospital settings and when contamination by viable mycobacteria is low.

## Figures and Tables

**Table 1:** Results for the setting and validation of bacteriological and molecular methods of *M. chimaera* detection. mMVT denotes modified Middlebrook 7H9 Broth; PBS-T phosphate-buffered saline added with 0.5% Tween 20; VT VersaTREK™ system; POS positive; NEG negative; TTD time to detection; SD standard deviation; N.A. not available.

	*s*7H11 (Solid Media)		VT Time To Detection		Real Time PCR	
Suspension (CFU/mL)	mMVT	PBS-T	mMVT (Mean Hours ± SD)	PBS-T (Mean Hours ± SD)	DNA Concentration Scale	Cq Mean
A (9 × 10^8^)	NEG	**POS**	67.2 ± 6.3	67.2 ± 6.3	N.A.	N.A.
B (9 × 10^7^)	NEG	**POS**	62.4 ± 8.7	64.8 ± 4.1	N.A.	N.A.
C (9 × 10^6^)	NEG	NEG	67.4 ± 2.5	89.6 ± 3.7	100 ng/µL	23.02
D (9 × 10^5^)	NEG	NEG	81.6 ± 2.4	113.6 ± 5	10 ng/µL	26.18
E (9 × 10^4^4)	NEG	NEG	104.2 ± 1.3	130.3 ± 3.8	1 ng/µL	29.34
F (9 × 10^3^)	NEG	NEG	137.5 ± 3.8	172.8 ± 4.8	100 pg/µL	32.78
G (9 × 10^2^)	NEG	NEG	156.4 ± 6.6	205.6 ± 5	10 pg/µL	36.17
H (9 × 10^1^)	NEG	NEG	200.9 ± 1.6	216 ± 4.8	NEG	NEG
I (9)	N.A.	NEG	259.5 ± 3.9	257.6 ± 5	NEG	NEG

**Table 2:** Specificity test of real-time PCR.

Bacterial Species	RtPCR Result	Bacterial Species	RtPCR Result
*Staphylococcus aureus*	NEG	*Mycobacterium abscessus*	NEG
*Francisella tularensis*	NEG	*Mycobacterium avium*	NEG
*Campylobacter fetus* subsp. *fetus*	NEG	*Mycobacterium avium* subsp. *avium*	NEG
*Yersinia pestis*	NEG	*Mycobacterium avium* subsp. *hominissuis*	NEG
*Nocardia* spp.	NEG	*Mycobacterium chelonae*	NEG
*Rhodococcus* spp.	NEG	*Mycobacterium intracellulare*	NEG *
*Corynebacterium* spp.	NEG	*Mycobacterium kansasii*	NEG
*Rhodococcus P2*	NEG	*Mycobacterium marinum*	NEG
*Escherichia coli*	NEG	*Mycobacterium microti*	NEG
*Brucella abortus*	NEG	*Mycobacterium* spp.	NEG
*Bacillus anthracis*	NEG	*Mycobacterium tuberculosis*	NEG

Asterisk indicates a weak signal of positivity (Ct > 38) in some replicates of *Mycobacterium intracellulare.* POS positive; NEG negative.

**Table 3:** Bacteriological and molecular methods for detection of *M. chimaera* in environmental simulates: 1000 mL (a, b, c, d, e) and 100 mL (a*, b*, c*, d*, e*) volume, respectively. POS positive; NEG negative; N.A. not available.

**Suspension 1000 mL**	**Stock Solution**	***s*7H11** **(Solid Media)**		**VT Time To Detection** **(Days)**		**RtPCR**
	**Expected Value (CFU/mL)**	**Result**	**Days**	**Result**	**Days**	**Cq Mean**
a	1.5 × 10^6^	POS	6	POS	2.5	21.18
b	1.5 × 10^5^	POS	6	POS	3.3	23.78
c	1.5 × 10^4^	POS	6	POS	4.1	26.55
d	1.5 × 10^3^	POS	9	POS	4.7	30.56
e	1.5 × 10^2^	POS	9	POS	11.5	35.39
**Suspension 100 mL**	**Stock Solution**	***s*7H11** **(Solid Media)**		**VT Time To Detection** **(Days)**		**RtPCR**
	**Expected Value** **(CFU/mL)**	**Result**	**Days**	**Result**	**Days**	**Cq Mean**
a*	1.5 × 10^6^	POS	7	POS	2.9	29.67
b*	1.5 × 10^5^	POS	7	POS	5	33.74
c*	1.5 × 10^4^	POS	7	POS	6.7	37.02
d*	1.5 × 10^3^	POS	7	POS	8.6	NEG
e*	1.5 × 10^2^	NEG	N.A.	POS	13.1	NEG

Real-time PCR on material obtained at the end of solid and the liquid culture confirmed the presence of *M. chimaera* DNA.

## Data Availability

Availability of data and materials and requests for materials should be addressed to the corresponding author.
